# Prediction of recurrence of ischemic stroke within 1 year of discharge based on machine learning MRI radiomics

**DOI:** 10.3389/fnins.2023.1110579

**Published:** 2023-05-04

**Authors:** Jianmo Liu, Yifan Wu, Weijie Jia, Mengqi Han, Yongsen Chen, Jingyi Li, Bin Wu, Shujuan Yin, Xiaolin Zhang, Jibiao Chen, Pengfei Yu, Haowen Luo, Jianglong Tu, Fan Zhou, Xuexin Cheng, Yingping Yi

**Affiliations:** ^1^Department of Medical Big Data Research Centre, The Second Affiliated Hospital of Nanchang University, Nanchang, China; ^2^School of Public Health, Jiangxi Provincial Key Laboratory of Preventive Medicine, Nanchang University, Nanchang, China; ^3^Department of Neurology, The Second Affiliated Hospital of Nanchang University, Nanchang, China; ^4^Biological Resource Center, The Second Affiliated Hospital of Nanchang University, Nanchang, China

**Keywords:** machine learning, radiomics, ischemic stroke, recurrence prediction, diffusion-weighted imaging

## Abstract

**Purpose:**

This study aimed to investigate the value of a machine learning-based magnetic resonance imaging (MRI) radiomics model in predicting the risk of recurrence within 1 year following an acute ischemic stroke (AIS).

**Methods:**

The MRI and clinical data of 612 patients diagnosed with AIS at the Second Affiliated Hospital of Nanchang University from March 1, 2019, to March 5, 2021, were obtained. The patients were divided into recurrence and non-recurrence groups according to whether they had a recurrent stroke within 1 year after discharge. Randomized splitting was used to divide the data into training and validation sets using a ratio of 7:3. Two radiologists used the 3D-slicer software to label the lesions on brain diffusion-weighted (DWI) MRI sequences. Radiomics features were extracted from the annotated images using the pyradiomics software package, and the features were filtered using the Least Absolute Shrinkage and Selection Operator (LASSO) regression analysis. Four machine learning algorithms, logistic regression (LR), Support Vector Classification (SVC), LightGBM, and Random forest (RF), were used to construct a recurrence prediction model. For each algorithm, three models were constructed based on the MRI radiomics features, clinical features, and combined MRI radiomics and clinical features. The sensitivity, specificity, and area under the receiver operating characteristic (ROC) curve (AUC) were used to compare the predictive efficacy of the models.

**Results:**

Twenty features were selected from 1,037 radiomics features extracted from DWI images. The LightGBM model based on data with three different features achieved the best prediction accuracy from all 4 models in the validation set. The LightGBM model based solely on radiomics features achieved a sensitivity, specificity, and AUC of 0.65, 0.671, and 0.647, respectively, and the model based on clinical data achieved a sensitivity, specificity, and AUC of 0.7, 0.799, 0.735, respectively. The sensitivity, specificity, and AUC of the LightGBM model base on both radiomics and clinical features achieved the best performance with a sensitivity, specificity, and AUC of 0.85, 0.805, 0.789, respectively.

**Conclusion:**

The ischemic stroke recurrence prediction model based on LightGBM achieved the best prediction of recurrence within 1 year following an AIS. The combination of MRI radiomics features and clinical data improved the prediction performance of the model.

## 1. Introduction

Stroke is a major chronic non-communicable disease that poses a serious health risk to the population. The disease is characterized by high morbidity, disability, mortality, recurrence, and economic burden. According to the global burden of disease (GBD), the lifetime risk of stroke in China is 39.3% for people over 25 years of age (Feigin et al., 2018). The 1-year recurrence rate after the first stroke ranges between 9.8–23.0% (Hobeanu et al., 2022; Li et al., 2022). Patients who experience a recurrent stroke are six times more likely to have another stroke in 10 years (Hardie et al., 2004). Recurrent strokes have a high rate of mortality and disability (Hankey et al., 2002). The disability rate of recurrent stroke patients is 8.49 times higher when compared with that of first stroke patients (Cucchiara et al., 2020). As a result, there is a need to predict the risk of recurrent strokes to improve the patient’s quality of life and reduce the mortality rate.

Currently, AIS are diagnosed using computed tomography (CT) and MRI (Phipps and Cronin, 2020). MRI sequences such as DWI and fluid-attenuated inversion recovery (FLAIR) are highly sensitive for the detection of ischemic strokes (Powers et al., 2019). Studies have shown that MRI may have an important role in predicting recurrence after the first AIS (Kauw et al., 2018; Jing et al., 2021; Fote et al., 2022). However, the manual evaluation of MRI images is subjective and does not fully observe all the underlying information in the image. Radiomics is increasingly being used to transform medical images into high-throughput quantitative features. These features could be used to predict treatment outcomes of various diseases (van Griethuysen et al., 2017), including the risk of recurrence following an AIS (Su et al., 2020). Nevertheless, it is important to acknowledge that apart from radiomics features, several clinical factors may also have an important role in the treatment outcomes of an AIS. Machine learning could be used to combine the radiomics features with high-risk clinical factors to develop predictive models for the development of recurrence following an AIS. Mainly because of the wide range of applications of machine learning in healthcare, there have been studies using machine learning algorithms for stroke diagnosis and prognosis prediction and they have shown very good performance (Heo et al., 2019; Murray et al., 2020).

Therefore in this study, we aimed to use DWI radiomics and clinical features to develop different machine-learning algorithms for predicting recurrence within 1 year following an AIS. The optimal predictive model could be used to facilitate the early diagnosis and treatment of AIS and hence improve survival while reducing the disease burden on the patients, carers, and society.

## 2. Materials and methods

### 2.1. Study population

This study was evaluated and granted by the Medical Research Ethics Committee of the Second Affiliated Hospital of Nanchang University (2018-05). Patients with MRI-diagnosed AIS were recruited from the Second Affiliated Hospital of Nanchang University from March 1, 2019, to March 5, 2021. All patients aged between 18–85 years who had DWI imaging within 48 h of admission for AIS and a National Institute of Health stroke scale (NIHSS) score of 15 or less were included in the study. Cancer patients and those with severe cardiac, pulmonary, and hepatic system diseases were excluded. In addition, the patients with major artifacts on the DWI were also excluded. A total of 612 patients were eventually included, all were followed up for 1 year to track whether the patients experienced a recurrence of stroke. The patients were divided into training and validation sets using a 7:3 randomized grouping ratio.

### 2.2. Data collection

#### 2.2.1. Relevant clinical data of the patients

The patient’s demographic characteristics (age and gender), history of previous diseases (e.g., hypertension, diabetes, atrial fibrillation, stroke, and ischemic heart disease), and alcohol and smoking history were extracted from the clinical medical records. In addition, the physical examination data (height, weight, and blood pressure) and laboratory test results (routine blood, liver function, kidney function, and coagulation function) were also extracted. Finally, the DWIs and the recurrence outcome variable within 1 year after hospital discharge were also collected.

#### 2.2.2. MRI image acquisition

All patients underwent a DWI brain imaging within 48 h of admission. All images were acquired on a GE 3.0 T MRI scanner, using a repetition time (TR) of 4,090 ms, an echo time (TE) of 98 ms, a flip angle of 180°, a scan field of view of 230 mm × 230 mm, a matrix of 192 × 192, 20 layers of a thickness of 6 mm and layer spacing of 1.3 mm, a *b*-value of 0, 1,000 s/mm2, and a duration of 30 s.

#### 2.2.3. Radiomics feature extraction

Two radiologists with 5 years of experience used the 3D slicer software (Version 4.13.0)^1^ to annotate the ischemic lesions on the DWI, respectively. The MDice, MIOU, and Hausdorff metrics were used to verify the consistency of the annotations. The consistency of the annotated volumes was evaluated using the Bland–Altman method and the intraclass correlation coefficient (ICC). If the ICC was less than 0.75, a third imaging specialist with a senior title was asked to label and verify the annotation. Subsequently, the first-order, morphological, and texture radiomics features were extracted from the annotated DWI images using the Pyradiomics package (van Griethuysen et al., 2017). The first-order and texture features were extracted from the original image, Gaussian Laplace filtered image (sigma = 1.0, 2.0, 3.0), and the wavelet-transformed image. For the wavelet-transformed images, all combinations of high-pass (H) and low-pass (L) filters were applied in all three dimensions, including LLH, LHL, LHH, HLL, HLH, HHL, HHL, HHH, LLL, with a group distance of 10.

The Pearson intergroup correlation coefficient was used for feature redundancy analysis. Features with correlation coefficients greater than 0.9 were excluded. The optimal radiomics features for the prediction of AIS were screened using LASSO regression with 10-fold cross-validation.

### 2.3. Construction and validation of the prediction model

Four machine learning algorithm models, including LR, SVC, LightGBM, and RF, were used to develop the prediction model for recurrent AIS. For each algorithm, three prediction models were constructed using the radiomics features, the clinical features, and the radiomics features combined with the clinical features. Training model using 10-fold cross-validation. The sensitivity, specificity, receiver operating characteristic (ROC) curve, and area under the curve (AUC) were used to compare the performance of the models. The clinical application value of the prediction models was evaluated using a calibration curve and a decision curve analysis (DCA).

### 2.4. Statistical analysis

The statistical package for social science (SPSS) software version 22.0 was used to analyze the data. The categorical data were summarized as frequency (%), and Fisher’s exact test was used to evaluate the differences between the recurrent AIS group and the non-recurrent AIS group. The continuous data were tested for normality using Kolmogorov–Smirnov test. The normally distributed data were summarized as mean ± standard deviation (x ± S), and the differences between the two groups were compared using the *t*-test. Conversely, the non-normally distributed data were described using median and upper and lower quartiles [M (P25, P75)], and comparisons between groups were made using the Mann–Whitney U test. For all statistical tests, a *p*-value below 0.05 was deemed statistically significant.

## 3. Results

### 3.1. Baseline analysis of data

A total of 612 patients, including 337 males and 275 females, were enrolled in this study. The average age of the patients was 63.9 ± 11.16 years. 67 (10.95%) patients were found to have had a recurrence of stroke at the 1-year follow-up. Univariate analysis showed that smoking history, ischemic heart disease history, stroke history, alkaline phosphatase, creatinine, prothrombin time, fibrinogen concentration, age, absolute neutrophil value, total protein, albumin, white blood cell count, and international labeling ratio were associated with stroke recurrence (*P* < 0.05), as shown in Table 1. Comparison of full baseline data in Supplementary Table 1.

**TABLE 1:** Comparison of certain clinical characteristics between the recurrence group and the non-recurrence group.

Variable	Non-recurrence group (*n* = 545)	Recurrence group (*n* = 67)	*χ^2^/t/z*	*p*
Age M (Q_25_, Q_75_)	63.499 (56, 72)	67.164 (56, 75)	−2.554	0.011
Smoking history [n (%)]			3.868	0.049
Yes	344 (63.469)	34 (50.746)		
No	201 (36.881)	33 (49.254)		
Stroke history [n (%)]			5.646	0.017
Yes	110 (20.183)	22 (32.836)		
No	435 (79.817)	45 (67.164)		
**Ischemic heart disease history [n (%)]**				
Yes	6 (1.101)	3 (4.478)	4.695	0.030
No	539 (98.899)	64 (95.522)		
Alkaline phosphatase [mean (SD)]	90.149 (24.971)	97.386 (32.255)	−2.157	0.031
Creatinine [mean (SD)]	77.621 (25.539)	89.11 (34.935)	−2.591	0.012
Prothrombin time [mean (SD)]	11.292 (1.238)	11.675 (2.140)	−2.161	0.031
Fibrinogen concentration [mean (SD)]	2.950 (0.846)	3.341 (1.221)	−2.528	0.014
Absolute neutrophil count [mean (SD)]	4.856 (2.111)	5.654 (3.084)	−2.045	0.044
Total protein [mean (SD)]	66.347 (5.109)	64.746 (5.257)	2.408	0.016
Albumin [mean (SD)]	37.864 (3.277)	36.660 (4.155)	2.270	0.026
White blood cell count [mean (SD)]	7.242 (2.350)	7.960 (3.207)	−2.254	0.025
International standard rate [mean (SD)]	0.979 (0.109)	1.013 (0.192)	−2.177	0.030

### 3.2. Image annotation consistency test

**FIGURE 1:**
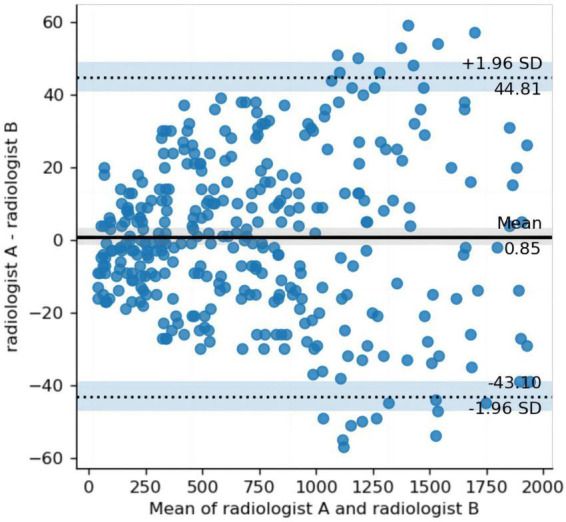
Bland–Altman plot comparing the differences in the volumes annotated by the two radiologists. The *y*-axis represents the difference (between radiologist A and radiologist B volumes), and the *x*-axis, the mean of radiologist A and radiologist B. Middle solid line and flanking dashed lines = means ± 1.96 standard deviation, respectively.

The mean MDice, MIOU, and Hausdorff distances for the annotations made by the two radiologists were 0.87, 0.77, and 4.36 mm, respectively. These results indicate a good agreement between the observers. Figure 1 illustrates the Bland–Altman plot for the volumes annotated by the two radiologists. The results of the Bland–Altman plot show that the difference in the annotated volume between the two observers ranged between +44.81 ml and −43.10 ml. 96.7% (*n* = 592) of the cases. The delineated volume was outside the limits of agreement (mean ± 1.96 SD) in 3.3% (*n* = 20) of cases. The mean ICC between the lesion volumes annotated by the two radiologists was 0.99 (95 CI: 0.99 to 1, *p* < 0.01), indicating a good agreement.

### 3.3. Extraction and screening of radiomics features

**FIGURE 2:**
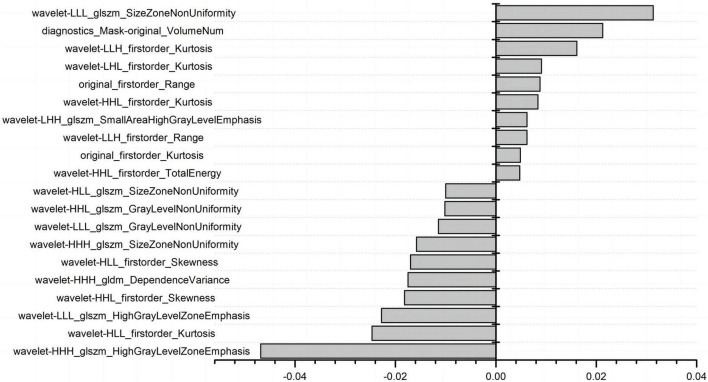
The 20 radiomics features extracted by LASSO.

A total of 1,037 radiomics features were extracted, including 198 histogram features, 14 morphological factor features, and 825 texture features. The texture features included 264 grayscale co-generation matrices (GLCM), 176 grayscale tour matrices (GLRLM), 176 grayscale size region matrices (GLSZM), 154 grayscale dependence matrices (GLDM), and 55 neighborhood grayscale difference matrices (NGTGM). The feature redundancy analysis (Supplementary Figure 1) resulted in 112 radiomics features. Figure 2 shows the final 20 features extracted by LASSO regression.

The interpretation of the imaging histology features can be retrieved from.^2^

### 3.4. Construction and validation of prediction models

The training and validation datasets consisted of 428 and 184 patients, respectively. In the validation set, the prediction accuracy of the 4 machine learning models for the radiomics features, clinical features, and the radiomics and clinical features combined are summarized in Table 2. The ROC for all four models is shown in Supplementary Figure 2. All four models achieved the highest prediction accuracy on the combined radiomics and clinical data, followed by the clinical and radiomics data. The LightGBM model based on data with three different features achieved the best prediction accuracy from all four models with a sensitivity, specificity, and AUC of 0.85, 0.805, and 0.789, respectively. The same model achieved a sensitivity of 0.7, a specificity of 0.799, and an AUC of 0.735 on the clinical data and a sensitivity of 0.65, a specificity of 0.671, and an AUC of 0.647 for the radiomics data.

**TABLE 2:** Prediction results of the four models with different data sets.

Model	Radiomics data			Clinical treatment data			Clinical treatment data with radiomics data		
	Sensitivity	Specificity	AUC (95% CI)	Sensitivity	Specificity	AUC (95% CI)	Sensitivity	Specificity	AUC (95% CI)
LR	0.55	0.646	0.613 (0.582, 0.639)	0.65	0.75	0.704 (0.663, 0.727)	0.8	0.768	0.764 (0.714, 0.783)
SVC	0.55	0.652	0.615 (0.579, 0.641)	0.65	0.774	0.711 (0.661, 0.735)	0.8	0.787	0.768 (0.743, 0.799)
LightGBM	0.65	0.671	0.647 (0.617, 0.689)	0.7	0.799	0.735 (0.724, 0.782)	0.85	0.805	0.789 (0.745, 0.814)
RF	0.6	0.652	0.622 (0.591, 0.656)	0.7	0.787	0.728 (0.691, 0.757)	0.8	0.793	0.772 (0.717, 0.811)

**FIGURE 3:**
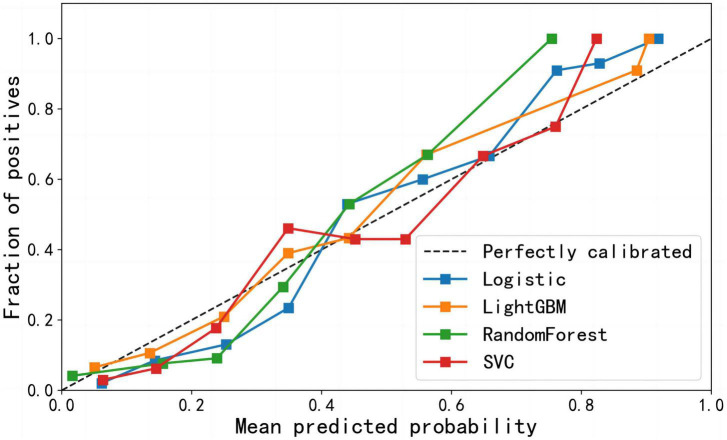
Calibration curve of four machine learning models.

**FIGURE 4:**
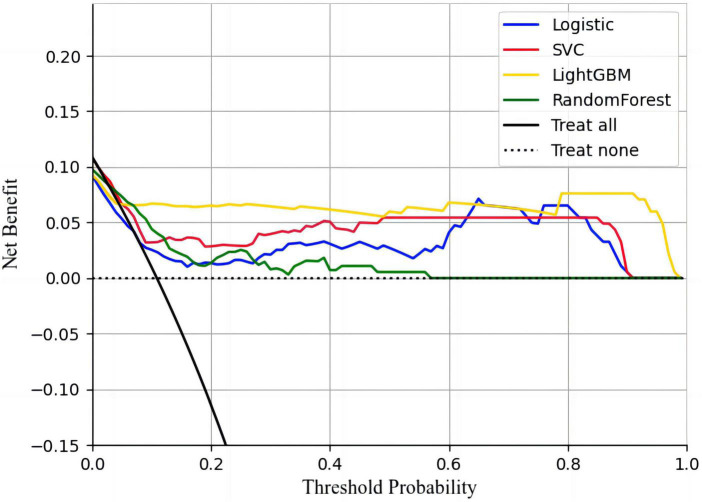
Four machine learning models decision curve analysis.

The calibration curves showed that all four models had a high calibration. The LightGBM model has the best discrimination. The specific performance is shown in Figure 3. The decision curves showed that all four machine learning models could improve decision-making under different decision thresholds as the models correctly predicted all AIS recurrences (Figure 4). However, the LightGBM and SVC models achieved the best gains.

### 3.5. Analysis of influencing factors based on the optimal model

**FIGURE 5:**
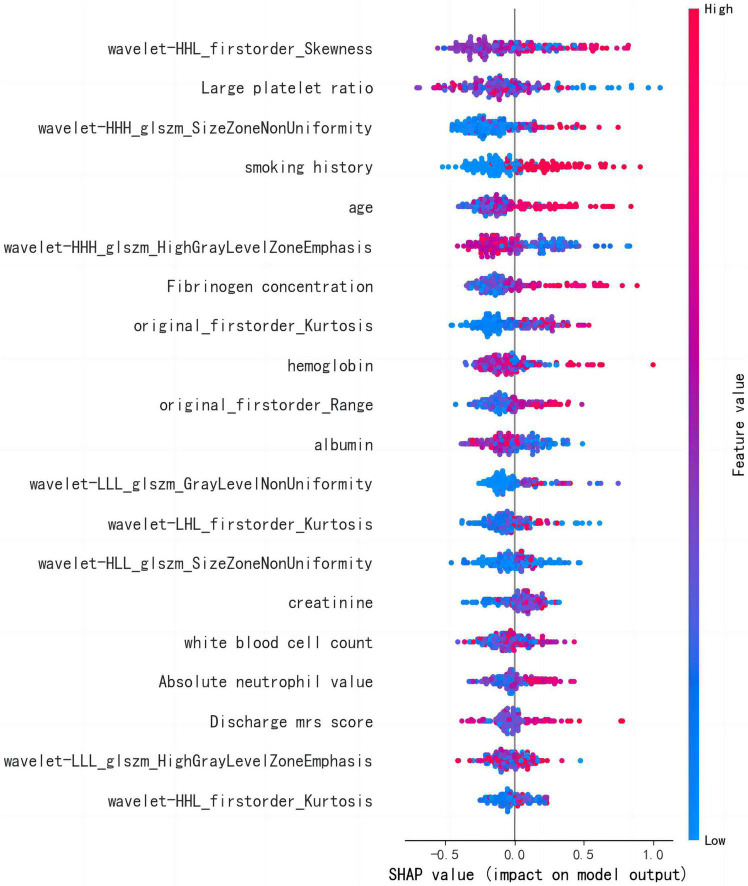
LightGBM model feature importance diagram.

The LightGBM algorithm achieved the best performance. The top 20 influencing factors in the LightGBM model are shown in Figure 5. Among these influencing factors, 10 were clinical diagnostic indicators and included hemoglobin, large platelet ratio, creatinine, white blood cell count, age, international standard ratio, alkaline phosphatase, fibrinogen concentration, albumin, and mean red blood cell volume. The other indicators included five first-order radiomics features and five textural features.

## 4. Discussion

Recurrence following an AIS leads to poor survival and quality of life. As a result, there is a need to develop models to predict the onset of AIS and hence minimize the morbidity and mortality of this disease. In this study, we developed four machine learning algorithms (LR, SVC, RF, and LightGBM) to predict recurrence following an AIS. Each algorithm was trained using clinical treatment data, MRI radiomics data, and combined clinical and MRI radiomics data.

The DWI sequence in MRI reflects the random Brownian motion of water molecules in the tissue. AIS causes water molecules to move from outside the cell to the inside of the cells. The DWI signal increases as the extracellular water volume decreases (Alegiani et al., 2019). Changes in DWI imaging can occur within minutes following an AIS (He et al., 2021). Within the first 6 h after the AIS onset, DWI is more sensitive in detecting the brain edema caused by AIS than non-contrast computed tomography (NCCT) (Nael et al., 2014). Therefore, for the development of this prediction model, we decided to use radiomics features extracted from DWI sequences.

Previous studies have extracted radiomics features from FLAIR and ADC, which can significantly improve the predictive performance of functional outcomes in AIS patients compared to clinical features only (Quan et al., 2021). Ramos et al.’s (2022) study found that combining imaging histology features and clinical data using deep learning methods significantly improved prognostic prediction for patients with AIS receiving reperfusion therapy. It is consistent with the results of this study that the prediction model using clinical with radiomics data has the best performance.

In our study, the LightGBM model achieved the best prediction performance. LightGBM is a fast, distributed, high-performance gradient-boosting framework based on a decision tree algorithm (Ke et al., 2017). Compared with the LR, SVC, and RF models, the LightGBM model fitted the data better. Light GBM mainly includes a Histogram-based decision tree algorithm, Leaf-Wise leaf growth strategy with depth restrictions, one-sided gradient sampling, and direct support for category features. Finding less accurate segmentation points using histogram discrete values may affect the results. However, in the test results of the unused dataset, the discretized segmentation points have little impact on the final accuracy of the algorithm, and the resulting results are even slightly better due to the fact that overfitting can be effectively prevented. In this study, the data features are large, and the LightGBM model no longer divides the data when dealing with high-dimensional data, thus reducing the computational effort, and the prediction results can be obtained quickly and efficiently using this algorithm for stroke recurrence prediction.

The LightGBM algorithm identified 10 important radiomics features for the prediction of recurrence in AIS. The imaging risk factors showed that higher stroke voxel differences and higher voxel kurtosis indicate a more severe disease and an increased risk of developing recurrent AIS. In addition, a lower texture characteristic skewness coefficient, lower HighGrayLevelZoneEmphasis, and higher Gray Level Non-Uniformity also increased the risk of recurrence.

Several clinical factors can influence the risk of developing recurrence after an AIS. In this study, we found that platelet large cell ratio (PLCR), smoking history, age, fibrinogen concentration, hemoglobin, albumin, creatinine, white blood cell count, absolute neutrophil value, and discharge the modified Rankin scale (mRS) score as important risk factors for the development of recurrence. Previous studies (Bergstrom et al., 2017; Felling et al., 2017; Wang et al., 2018) also identified age and years of smoking as risk factors for recurrence. Furthermore, studies have found (Mccabe et al., 2021) that elevated fibrinogen levels can increase plasma viscosity and promote platelet aggregation. Changes in the adhesion of the body’s red blood cells and endothelial cells can also stimulate the proliferation of smooth muscle cells, leading to endothelial damage and an increased risk of recurrence. A retrospective cohort study in Korea (Chang et al., 2020) showed that low hemoglobin could increase the risk of AIS recurrence. A prospective cohort study (Wu et al., 2021) that included white blood cell count and neutrophil in the low-grade inflammation (LGI) score, and showed that an increased LGI score was associated with an increased risk of stroke recurrence. There is also study (Zhu et al., 2018) showing that the coexistence of neutrophils and intracranial artery stenosis is associated with an increased risk of stroke recurrence. Serum albumin is a marker related to the patient’s nutritional status and inflammation (Capes et al., 2001). The synthesis of albumin is inhibited in malnourished patients (Pandian et al., 2011). Studies have shown that the risk of AIS recurrence is reduced by 14.6% for every 1 g/L increase in serum albumin levels (Chakraborty et al., 2013). The blood creatinine reflects the degree of renal impairment and can lead to increased blood pressure when renal function decreases. AIS guidelines now support the inclusion of blood creatinine levels in AIS recurrence prediction models (Meschia et al., 2014). Studies have also shown that the mRS score at discharge is a risk factor and higher mRS scores represent a more severe disease. Patients with mild ischemic stroke with high mRS scores may require more stringent control of risk factors after discharge. However, converse to previous studies, our findings indicate that the lower the PLCR, the lower the risk of developing stroke recurrence (Verma et al., 2020). Therefore additional studies are recommended to validate this finding.

This study has some limitations that have to be acknowledged. All the patients in this study were recruited from a single center, potentially introducing selection bias. Therefore larger multicenter studies are required to validate the model. The radiomics model was trained on 2-dimensional (2D) images instead of volumetric images. As a result, useful radiomics features between the 3D layers may have been omitted. Previous studies have shown that the 3D stereoscopic shape of AIS lesions could be used to predict recurrence (Frindel et al., 2015). Future studies should make use of 3D images to train the model.

## 5. Conclusion

The LightGBM model based on radiomics features extracted from DWI and clinical data achieved the best performance for predicting recurrence following an AIS. This model could provide a non-invasive tool for clinicians to assess the risk of recurrence following an AIS and hence improve the monitoring of high-risk patients. However, although this model was trained and validated on a relatively large dataset, further multicenter studies are required to validate the performance of this model.

## Data availability statement

The datasets presented in this article are not readily available because raw data involving sensitive personal information. Requests to access the datasets should be directed to YY, yyp66@126.com.

## Ethics statement

The studies involving human participants were reviewed and approved by the Ethics Committee of the Second Affiliated Hospital of Nanchang University. The patients/participants provided their written informed consent to participate in this study.

## Author contributions

JLiu and YW carried out the research, drafted the original manuscript, analyzed the data, and built the prediction models. YW, WJ, MH, YC, JLi, BW, SY, XZ, and JC collected the data. PY and HL provided technical support. JT, FZ, and XC provide clinical guidance. YY contributed to the design of the experiment and revised the manuscript. All authors contributed to the article and approved the submitted version.

## Footnotes

1
https://www.slicer.org

2
https://pyradiomics.readthedocs.io/en/latest/index.html
