# Supplementary figures and images for: Prediction of recurrence of ischemic stroke within 1 year of discharge based on machine learning MRI radiomics
Source: Front Neurosci. 2023 May 4;17:1110579. doi: 10.3389/fnins.2023.1110579 (PMC10192708; doi:10.3389/fnins.2023.1110579)

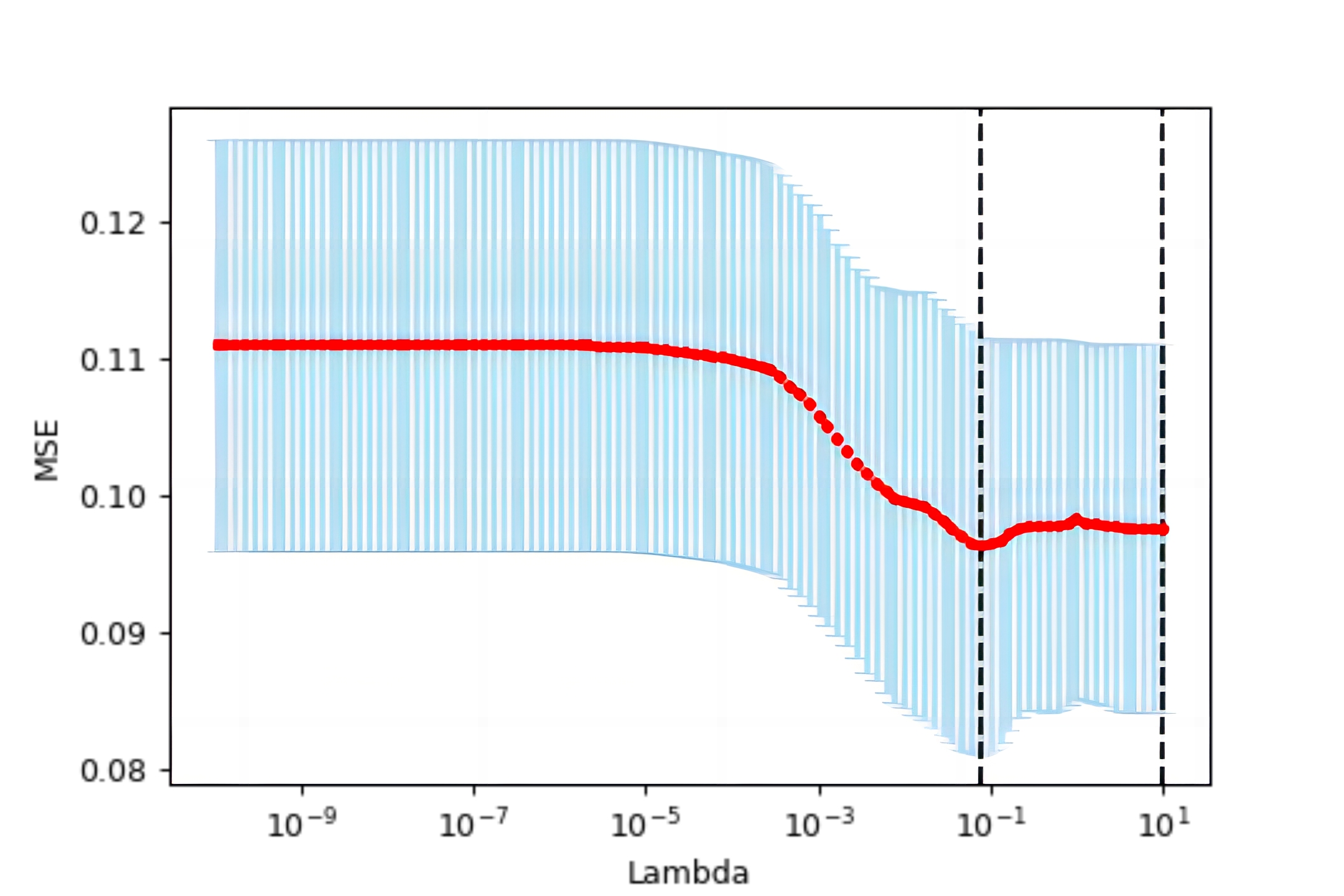

Supplement: Supplementary file 1 [file Data_Sheet_1.ZIP › Supplementary Figure 1(A).jpg]

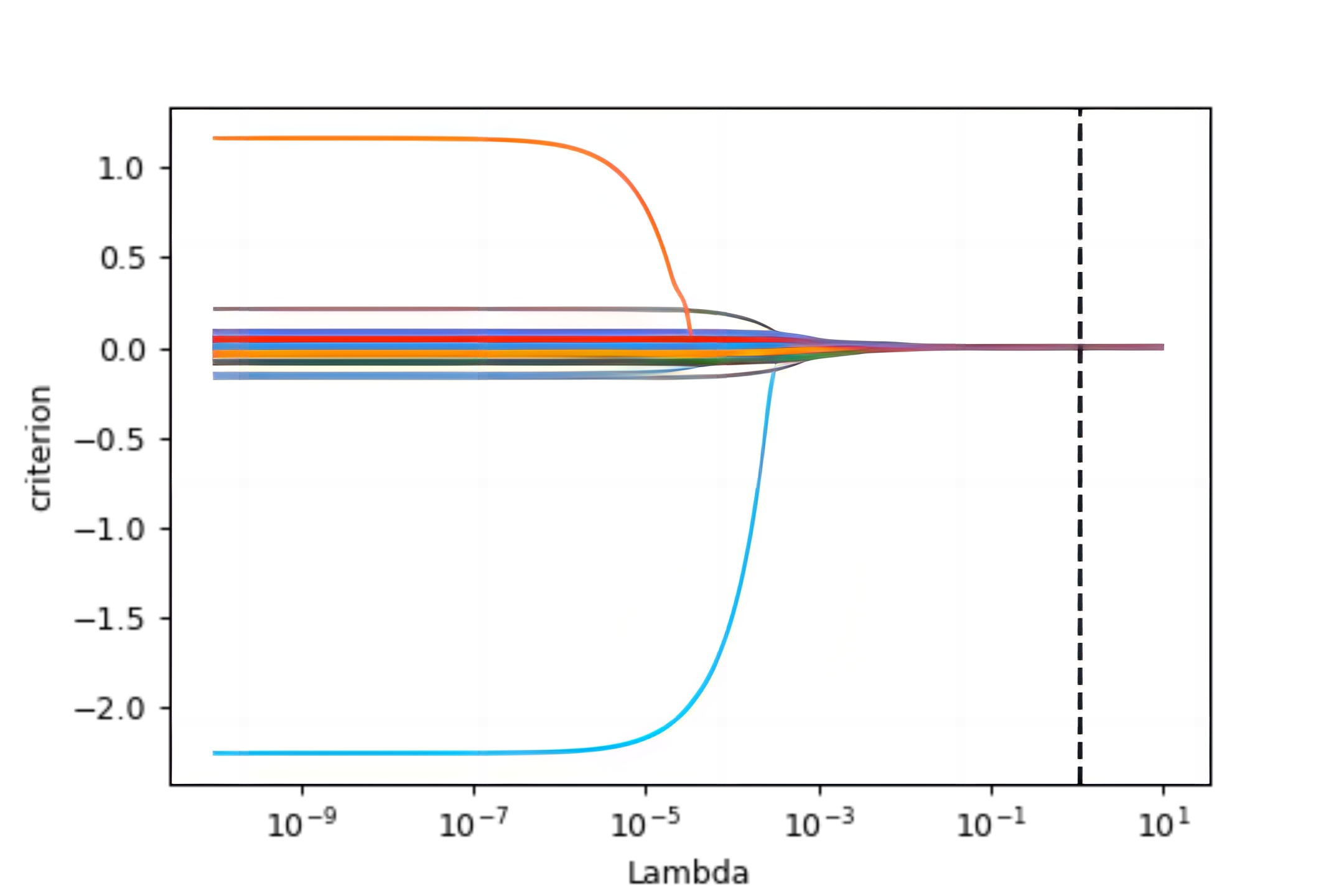

Supplement: Supplementary file 1 [file Data_Sheet_1.ZIP › Supplementary Figure 1(B).jpg]

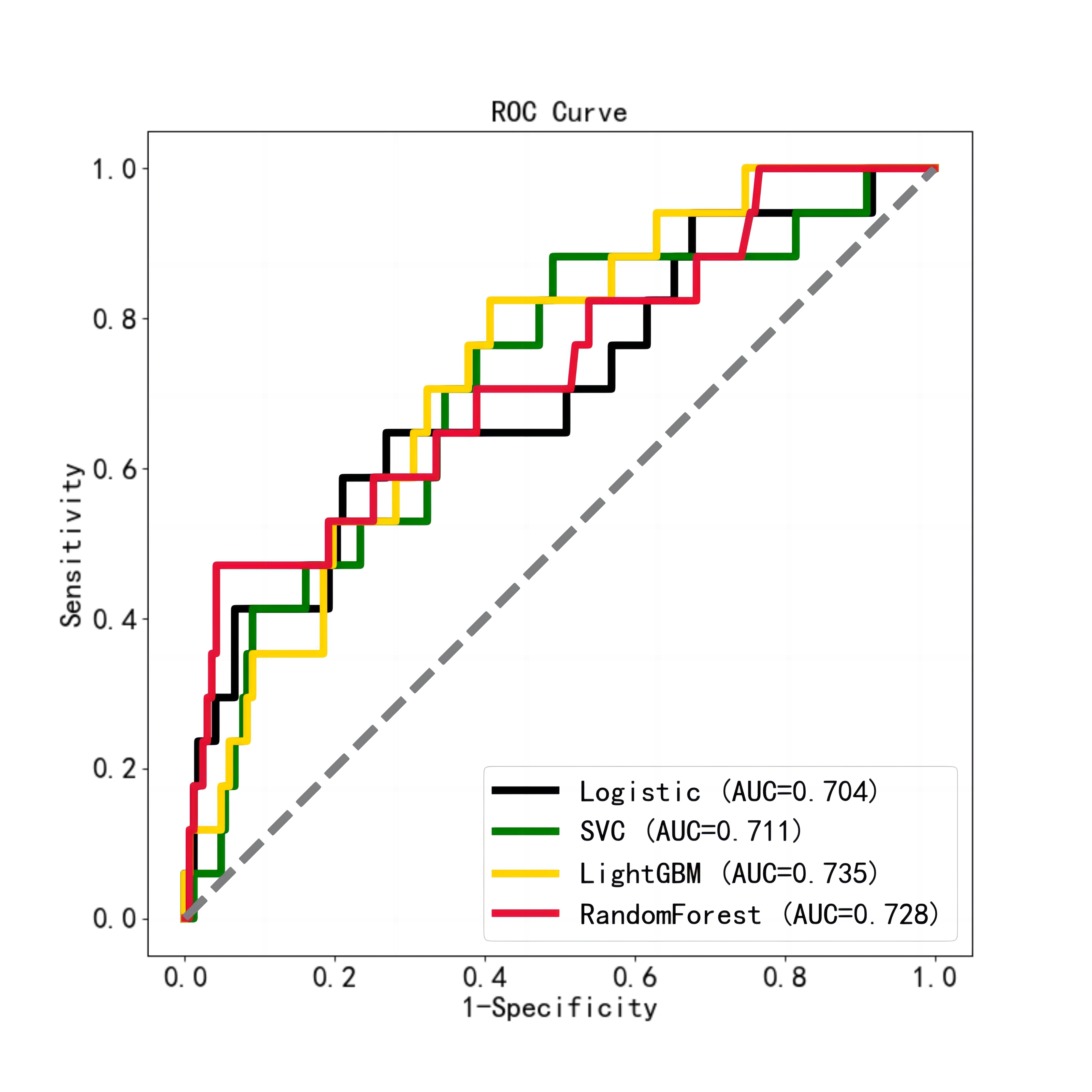

Supplement: Supplementary file 2 [file Data_Sheet_2.ZIP › Supplementary Figure 2(A).jpg]

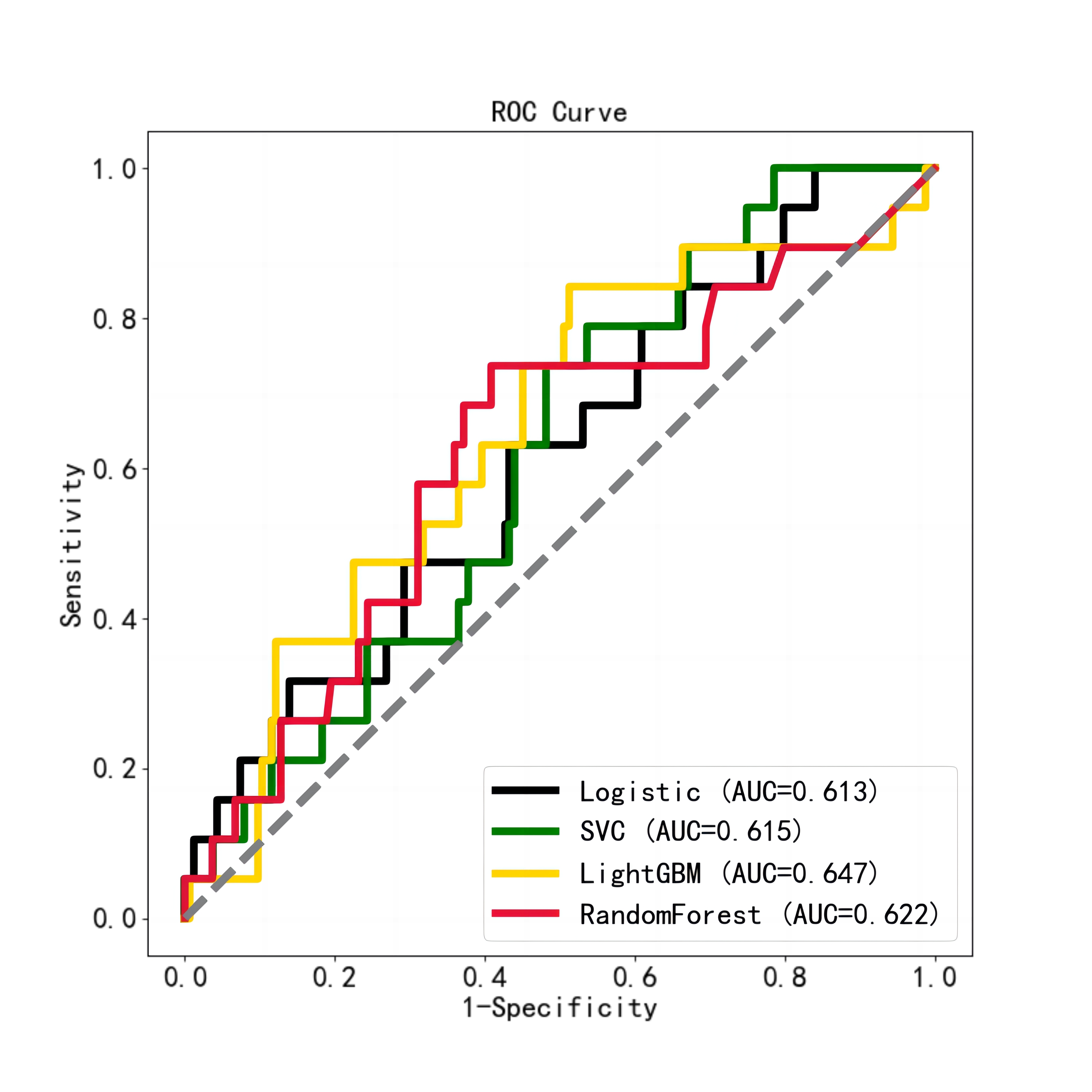

Supplement: Supplementary file 2 [file Data_Sheet_2.ZIP › Supplementary Figure 2(B).jpg]

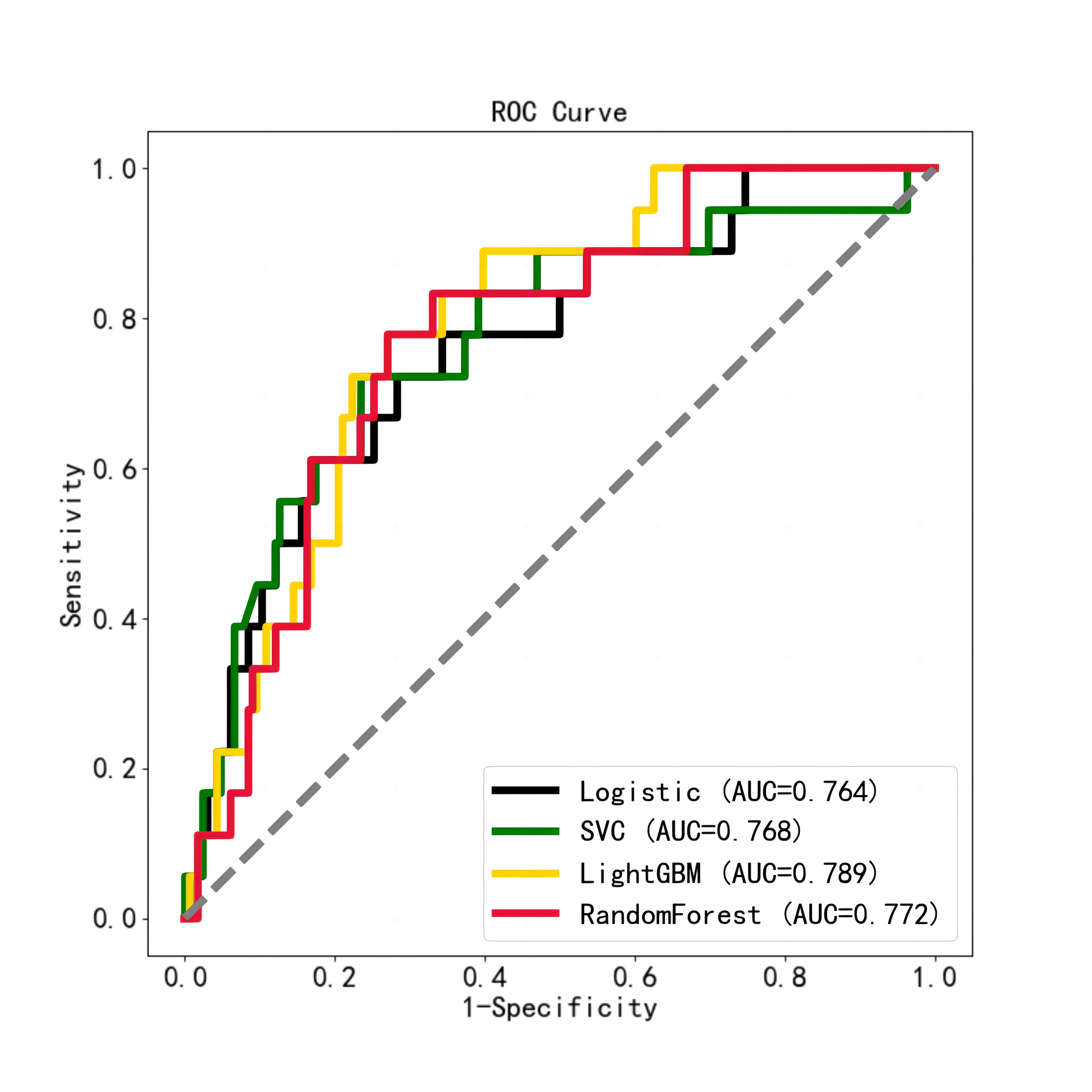

Supplement: Supplementary file 2 [file Data_Sheet_2.ZIP › Supplementary Figure 2(C).jpg]
