# Supplementary material for: Prediction of recurrence of ischemic stroke within 1 year of discharge based on machine learning MRI radiomics
Source: Front Neurosci. 2023 May 4;17:1110579. doi: 10.3389/fnins.2023.1110579 (PMC10192708; doi:10.3389/fnins.2023.1110579)
Supplement: Supplementary file 3 [file Table_1.DOCX]

Supplementary Material

Prediction of recurrence of ischemic stroke within 1 year of discharge based on machine learning MRI radiomics

Jianmo Liu^†^, Yifan Wu^†^, Weijie Jia, Mengqi Han, Yongsen Chen, Jingyi Li, Bin Wu, Shunjuan Yin, Xiaolin Zhang, Jibiao Chen, Pengfei Yu, Haowen Luo, Yingping Yi^*^

*** Correspondence:** Yingping Yi: yyp66@126.com

**Supplementary Table 1**: Comparison of all clinical features between the recurrence group and non-recurrence group.

| Variable | non-recurrence group  (n=545) | recurrence group  (n=67) | χ^2^/t/z | p |
| --- | --- | --- | --- | --- |
| Age M (Q_25_, Q_75_) | 63.499 (56, 72) | 67.164 (56, 75) | -2.554 | 0.011 |
| Sex |  |  | 0.300 | 0.584 |
| Male | 298(54.679) | 39(58.209) |  |  |
| Female | 247(45.321) | 28(41.791) |  |  |
| Smoking history, n (%) |  |  | 3.868 | 0.049 |
| Yes | 344(63.469) | 34(50.746) |  |  |
| No | 201(36.881) | 33(49.254) |  |  |
| Diabetes history, n (%) |  |  | 0.590 | 0.442 |
| Yes | 170(31.193) | 24(35.821) |  |  |
| No | 375(68.807) | 43(64.179) |  |  |
| Hypertension history, n (%) |  |  | 1.420 | 0.233 |
| Yes | 385(70.642) | 52(77.612) |  |  |
| No | 160(29.358) | 15(22.388) |  |  |
| Stroke history, n (%) |  |  | 5.646 | 0.017 |
| Yes | 110(20.183) | 22(32.836) |  |  |
| No | 435(79.817) | 45(67.164) |  |  |
| Ischemic heart disease history, n (%) |  |  |  |  |
| Yes | 6(1.101) | 3(4.478) | 4.695 | 0.030 |
| No | 539(98.899) | 64(95.522) |  |  |
| Height, mean (SD) | 164.240 (7.410) | 166.169 (7.226) | 3.594 | 0.058 |
| weight, mean (SD) | 62.992 (10.863) | 64.693 (12.764) | 1.263 | 0.262 |
| Diastolic pressure, mean (SD) | 84.770  (13.622) | 81.896 (12.001) | 2.712 | 0.100 |
| Systolic pressure, mean (SD) | 146.296 (23.060) | 146.284 (21.763) | 0.0 | 0.997 |
| Alkaline phosphatase, mean (SD) | 90.149 (24.971) | 97.386 (32.255) | -2.157 | 0.031 |
| Creatinine, mean (SD) | 77.621 (25.539) | 89.11 (34.935) | -2.591 | 0.012 |
| Prothrombin time, mean (SD) | 11.292 (1.238) | 11.675 (2.140) | -2.161 | 0.031 |
| Creatine kinase, mean (SD) | 118.402 (151.849) | 98.793 (63.811) | 1.009 | 0.316 |
| Creatine kinase isoenzyme, mean (SD) |  |  |  |  |
| Fibrinogen concentration, mean (SD) | 2.950(0.846) | 3.341(1.221) | -2.528 | 0.014 |
| Absolute Neutrophil count, mean (SD) | 4.856(2.111) | 5.654(3.084) | -2.045 | 0.044 |
| Platelet count, mean (SD) | 210.579 (60.487) | 222.672 (84.742) | 2.146 | 0.143 |
| Platelet ratio, mean (SD) | 32.357(8.535) | 30.603(9.353) | 2.415 | 0.121 |
| Mean corpuscular volume, mean (SD) | 91.229(5.921) | 90.573(8.079) | 0.639 | 0.525 |
| Homocysteine, mean (SD) | 15.803(8.835) | 16.024(6.634) | 0.035 | 0.851 |
| Total protein, mean (SD) | 66.347(5.109) | 64.746(5.257) | 2.408 | 0.016 |
| Albumin, mean (SD) | 37.864(3.277) | 36.660(4.155) | 2.270 | 0.026 |
| Hemoglobin, mean (SD) | 134.208 (16.553) | 129.791 (22.246) | 1.561 | 0.123 |
| White blood cell count, mean (SD) | 7.242(2.350) | 7.960(3.207) | -2.254 | 0.025 |
| International standard rate, mean (SD) | 0.979(0.109) | 1.013(0.192) | -2.177 | 0.030 |

**Supplementary Figure 1:** Minimal absolute shrinkage and LASSO regression analysis to screen for imaging histological

**Supplementary Figure 2:** ROC for the LR, SVC, LightGBM, and RF models based on radiomics data(A), clinical treatment data, (B) and clinical treatment data with radiomics data(C).
